# Metal artifacts and artifact reduction of neurovascular coils in photon-counting detector CT versus energy-integrating detector CT — in vitro comparison of a standard brain imaging protocol

**DOI:** 10.1007/s00330-022-09073-y

**Published:** 2022-08-20

**Authors:** Niclas Schmitt, Lena Wucherpfennig, Lukas T. Rotkopf, Stefan Sawall, Hans-Ulrich Kauczor, Martin Bendszus, Markus A. Möhlenbruch, Heinz-Peter Schlemmer, Dominik F. Vollherbst

**Affiliations:** 1 Department of Neuroradiology, Heidelberg University Hospital, Im Neuenheimer Feld 400, 69120 Heidelberg, Germany; 2 Department of Diagnostic and Interventional Radiology, Heidelberg University Hospital, Im Neuenheimer Feld 420, 69120 Heidelberg, Germany; 3 Division of Radiology, German Cancer Research Center (DKFZ), Im Neuenheimer Feld 280, 69120 Heidelberg, Germany; 4 Division of X-Ray Imaging and Computed Tomography, German Cancer Research Center (DKFZ), Im Neuenheimer Feld 280, 69120 Heidelberg, Germany

**Keywords:** Metal artifact reduction, Coils, Photon-counting CT, Aneurysm, Brain imaging

## Abstract

**Objectives:**

Photon-counting detector computed tomography (PCD-CT) is a promising new technique for CT imaging. The aim of the present study was the in vitro comparison of coil-related artifacts in PCD-CT and conventional energy-integrating detector CT (EID-CT) using a comparable standard brain imaging protocol before and after metal artifact reduction (MAR).

**Methods:**

A nidus-shaped rubber latex, resembling an aneurysm of the cerebral arteries, was filled with neurovascular platinum coils and inserted into a brain imaging phantom. Image acquisition and reconstruction were repeatedly performed for PCD-CT and EID-CT (*n* = 10, respectively) using a standard brain imaging protocol. Moreover, linear interpolation MAR was performed for PCD-CT and EID-CT images. The degree of artifacts was analyzed quantitatively (standard deviation in a donut-shaped region of interest) and qualitatively (5-point scale analysis).

**Results:**

Quantitative and qualitative analysis demonstrated a lower degree of metal artifacts in the EID-CT images compared to the total-energy PCD-CT images (e.g., 82.99 ± 7.89 Hounsfield units (HU) versus 90.35 ± 6.28 HU; *p* < 0.001) with no qualitative difference between the high-energy bin PCD-CT images and the EID-CT images (4.18 ± 0.37 and 3.70 ± 0.64; *p* = 0.575). After MAR, artifacts were more profoundly reduced in the PCD-CT images compared to the EID-CT images in both analyses (e.g., 2.35 ± 0.43 and 3.18 ± 0.34; *p* < 0.001).

**Conclusion:**

PCD-CT in combination with MAR have the potential to provide an improved option for reduction of coil-related artifacts in cerebral imaging in this in vitro study.

**Key Points:**

• *Photon-counting detector CT produces more artifacts compared to energy-integrating detector CT without metal artifact reduction in cerebral in vitro imaging after neurovascular coil-embolization*.

• *Spectral information of PCD-CT provides the potential for new post-processing techniques, since the coil-related artifacts were lower in PCD-CT images compared to EID-CT images after linear interpolation metal artifact reduction in this in vitro study*.

## Introduction

Computed tomography (CT) of the brain is still essential for diagnostics and treatment planning for a large number of brain disorders, for example detection of acute intracranial hemorrhage or ischemic stroke [1–3]. Besides the limitations of radiation exposure and insufficient soft tissue contrast, CT imaging may also be impeded by substantial artifacts in patients with metallic implants, in particular imaging after endovascular platinum coil-embolization, e.g., of intracranial aneurysms [4, 5]. Endovascular coil-embolization serves besides neurosurgical clipping as the first-line therapy for the treatment of cerebral aneurysms while both methods bear the low risk of periprocedural hemorrhage [6–8]. Since these coil-associated artifacts may cause a limited diagnostic value of subsequent CT recordings, especially for detection of acute cerebral hemorrhage or hypodense ischemic stroke areas surrounding the endovascular platinum coils, as well as the evaluation of aneurysm reperfusion after neurovascular coil-embolization, several acquisition and post-processing techniques have been introduced over the past years to improve image quality and reduce artifacts in clinical use [4, 5, 9–13]. One promising new technique is the photon-counting detector CT (PCD-CT). Compared to the energy-integrating detectors (EID) in currently used clinical CT scanners, this new technology enables the direct conversion of x-ray photons into electronic signals without requiring the intermediate step of converting photons into visible light, as in scintillators. PCD-CT can bring the advantage of a reduced radiation exposure, increased spatial resolution, correction of beam-hardening artifacts, and use of alternative contrast agents while creating opportunities for quantitative imaging [14]. Moreover, PCD-CT inherently provides spectral information which further has the potential of an improved metal artifact reduction (MAR) [15]. To date, there is only a limited number of studies available, investigating metal-related imaging artifacts as well as options for MAR in PCD-CT [15–18]. Most of these studies focus on orthopedic or dental metallic implants in bone imaging, while so far there is no study available investigating artifacts of endovascular platinum coils in cerebral imaging and compares the potential of both CT techniques for subsequent MAR using the same artifact reduction approach.

The aim of the present study was the systematic assessment of imaging artifacts caused by neurovascular platinum coils as well as the effect of MAR in PCD-CT compared to EID-CT in an experimental in vitro model using a standard brain imaging protocol.

## Materials and methods

### Preparation of the in vitro model

**Fig. 1:**
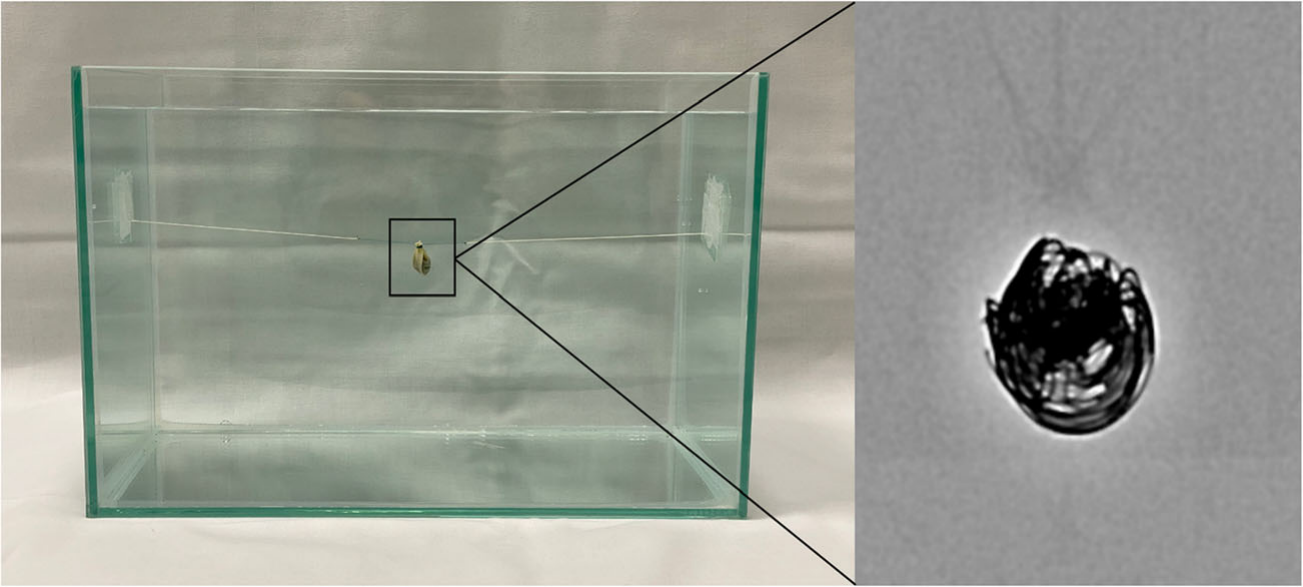
Schematic illustration of the experimental in vitro model, resembling a platinum coil–embolized aneurysm of the cerebral arteries, within the brain imaging phantom with an average attenuation similar to brain tissue. The in vitro model was fixed at the center of the imaging phantom using thin surgical threats

An experimental in vitro model, resembling an intracranial aneurysm of the cerebral arteries, was specifically designed for investigation of the present study. In brief, a nidus-shaped rubber latex with a diameter of 7 mm was filled with detachable platinum coils which are commonly used for the treatment of cerebral aneurysms in clinical practice using an Excelsior SL-10 microcatheter (Stryker Neurovascular): 1 × 6 mm × 20 cm Target XL 360 Soft coil; 1 × 6 mm × 15 cm Target 360 Soft; 2 × 5 mm × 10 cm Target 360 Soft; 2 × 3 mm × 10 cm Target 360 Ultra; and 1 × 2 mm × 4 cm Target 360 Nano (Stryker Neurovascular; respectively). After preparation, the experimental in vitro model was inserted into a custom-made brain imaging phantom with an average attenuation similar to brain tissue (mean 33.04 ± 0.86 Hounsfield units (HU)) — measured on the EID-CT images, consisting of saline (NaCl 0.9%) mixed with contrast medium (Imeron 300, Bracco Imaging GmbH) [19]. The in vitro model had a fixed placement at the center of the imaging phantom with minimal artificial movement between each scan. However, there was no movement between the corresponding PCT-CD and EID-CT scans to ensure reliable comparability. To reduce a possible influence caused by artificial air bubbles within the in vitro model, we inserted small holes to the natural rubber latex after insertion to the imaging phantom. This procedure ensured that the residual amount of air contained in the in vitro model was replaced by the mixture of saline and contrast medium of the imaging phantom. A schematic illustration of the imaging phantom is provided in Fig. 1.

### PCD-CT scanner system and imaging

Image acquisition of all CT images was performed on an experimental prototype whole-body PCD-CT scanner (SOMATOM CounT; Siemens Healthineers) at the German Cancer Research Center (DKFZ). In this CT system, the PCD is integrated in a dual-source system that also houses a conventional EID, while detailed technical information on the used CT scanner have been recently described by Yu et al [20]. For the present study, the Macro imaging mode was used that provides a detector pixel size similar to the EID. Settings for EID- and PCD-CT were similar to the standard brain imaging protocol of our institution. Image acquisition was performed at a tube voltage of 120 kV, as used in clinical routine. For PCD-CT, the low-energy threshold was set at 25 keV and high-energy threshold at 70 keV. Detailed information on the individual acquisition protocols are provided in Table 1. Each ten scans were conducted consecutively for PCD-CT and EID-CT, respectively.

**Table 1:** Settings of the PCD-CT and EID-CT acquisition protocols

	PCD-CT	EID-CT
Tube voltage	120 kV	120 kV
Tube current	60 mAs	60 mAs
Low-energy threshold/high-energy threshold	25/70 keV	
Collimation	32 × 0.5 mm	32 × 0.6 mm
Pitch	0.6	0.6
Reconstruction kernel	B30f	B30f
Slice thickness	1 mm	1 mm

CT scan protocols and reconstruction parameters of the photon-counting detector CT (PCD-CT) in Macro mode and the energy-integrating detector CT (EID-CT)

### Image reconstruction and post-processing

Image reconstruction of all scans was conducted with the ReconCT prototype software (version 14.0.1.45000; Siemens Healthineers) with a slice thickness of 1 mm and B30f brain imaging kernel. To ensure an adequate comparability between PCD-CT and EID-CT images, a weighted filtered back projection algorithm was used for reconstruction. In addition, PCD-CT scans were reconstructed as total-energy images (25–120 keV), low-energy bin images (25–70 keV), and high-energy bin images (70–120 keV). An overview of the individual reconstruction parameters is demonstrated in Table 1. After image reconstruction, linear interpolation MAR was applied to the total-energy PCD-CT and EID-CT images as described by Kalender et al [21]

### Quantitative image analysis

Quantitative image analysis was conducted with the Medical Imaging Interaction Toolkit (MITK; German Cancer Research Center (DKFZ)) [22].

As described previously, a special feature of the MITK software allowed us to place a standardized region of interest (ROI) with a donut-shaped configuration adjacent to and surrounding the center of the in vitro model of each scan [23–25]. This procedure allowed us the measurement of the surrounding artifacts while the coils were excluded from the analysis. The inner diameter of the donut-shaped ROI was 9 mm while the outer diameter was 108 mm. To ensure the consideration of artifacts of different positions, three separate ROIs were placed on the three central image slices and the standard deviation (SD) of the CT-values was calculated to assess the degree of artifacts. Manual adjustment of window width (W) and level/center (L) was allowed for an adequate ROI placement. Quantitative analysis was performed for the PCD-CT and EID-CT scans before and after MAR, focusing on the total-energy images of the PCD-CT scans (25–120 keV).

### Qualitative image analysis

Qualitative analysis of the degree of coil-related artifacts in PCD-CT and EID-CT scans before and after MAR was performed by two different readers (reader 1 with 4 years and reader 2 with 5 years of experience in diagnostic imaging) on a CENTRICITY PACS 4.0 workstation (GE Healthcare), both blinded to the type of detector and image reconstruction. Moreover, qualitative analysis for PCD-CT scans without MAR was conducted for total-energy images (25–120 keV), low-energy bin images (25–70 keV), and high-energy bin images (70–120 keV) while after MAR we focused on the total-energy PCD-CT images. All images were reviewed in the axial plane with standard brain window settings (W: 80 HU, L: 40 HU) while window adjustment was not allowed. The degree of coil-related artifacts was graded by a 5-point scale: (1) no artifacts, (2) minor artifacts, (3) moderate artifacts, (4) marked artifacts, (5) severe artifacts. To further improve the quality of the image analysis, a second read by both readers was performed after 6 weeks.

### Statistics

GraphPad Prism software (version 9.2.0) was used for statistical analysis. Results of the quantitative image analysis are provided as mean SD ± SD while results of the qualitative analysis are shown as mean score ± SD.

To evaluate statistical differences between the individual study groups before and after MAR, the Wilcoxon matched-pairs signed rank test was conducted. Moreover, the Kruskal-Wallis test and Dunn’s test for multiple comparisons using statistical hypothesis testing were performed for qualitative analysis.

Cohen’s *κ* coefficient was calculated to assess the inter-reader and intra-reader agreement of the qualitative analysis [26]. The *κ* values were interpreted as follows: ≤ 0.20, no agreement; 0.21–0.39, minimal agreement; 0.40–0.59, weak agreement; 0.60–0.79, moderate agreement; 0.80–0.90, strong agreement; and ≥ 0.90, almost perfect agreement. The level of statistical significance was defined as *p* < 0.05 [27].

## Results

**Fig. 2:**
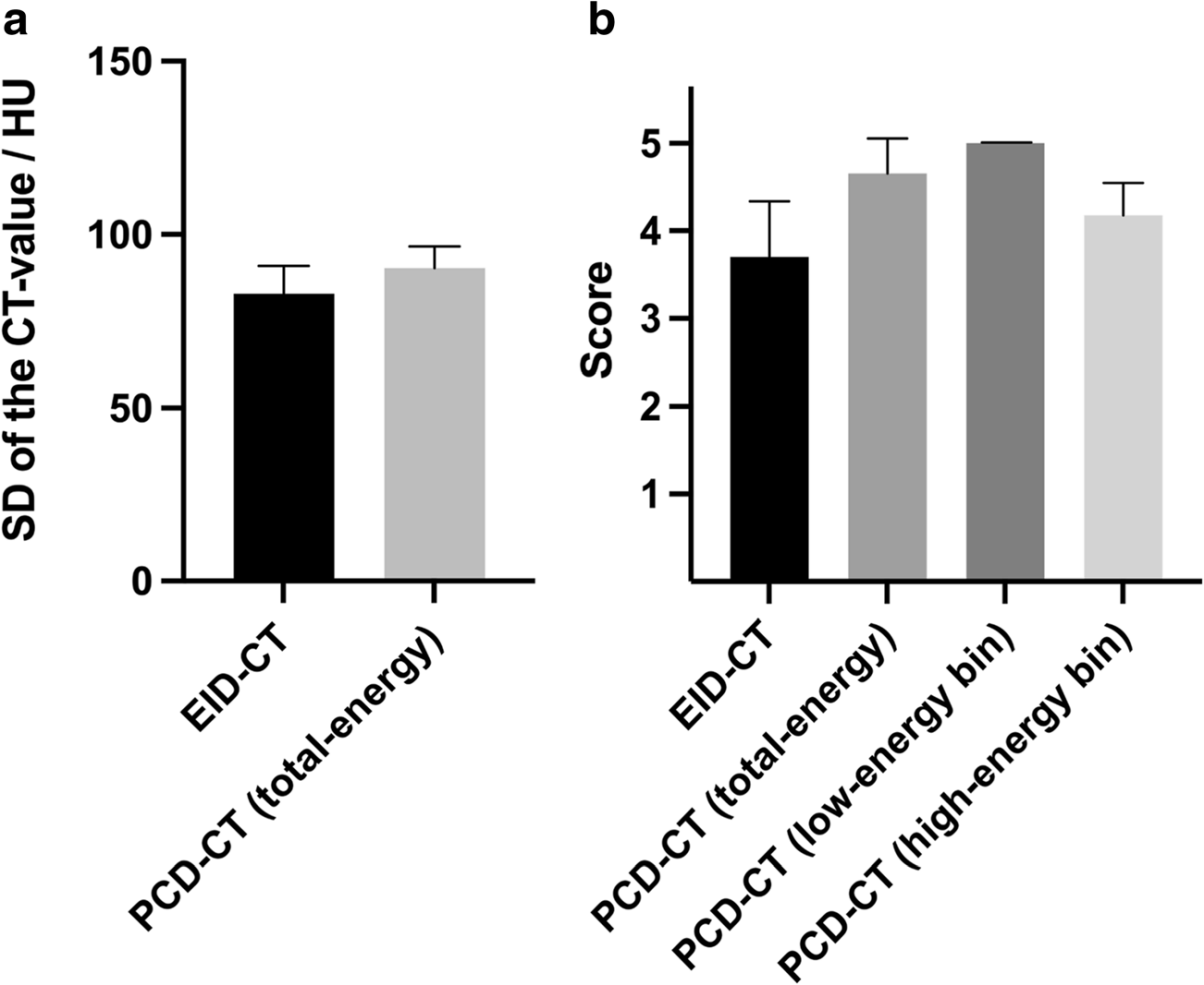
Illustration of the results of the quantitative and qualitative image analysis without metal artifact reduction. Quantitative analysis (**a**) using a donut-shaped region of interest demonstrated a higher degree of coil-related artifacts in the total-energy PCD-CT images compared to the EID-CT images. Qualitative analysis (**b**) using a 5-point scale revealed the highest degree of artifacts in the low-energy bin PCD-CT images and the lowest degree in the high-energy bin PCD-CT images. There was no statistical difference between the EID-CT and the high-energy bin PCD-CT images. CT, computed tomography; EID-CT, energy-integrating computed tomography; HU, Hounsfield units; PCD-CT, photon-counting detector computed tomography; SD, standard deviation. Bars: mean; whiskers: SD

**Fig. 3:**
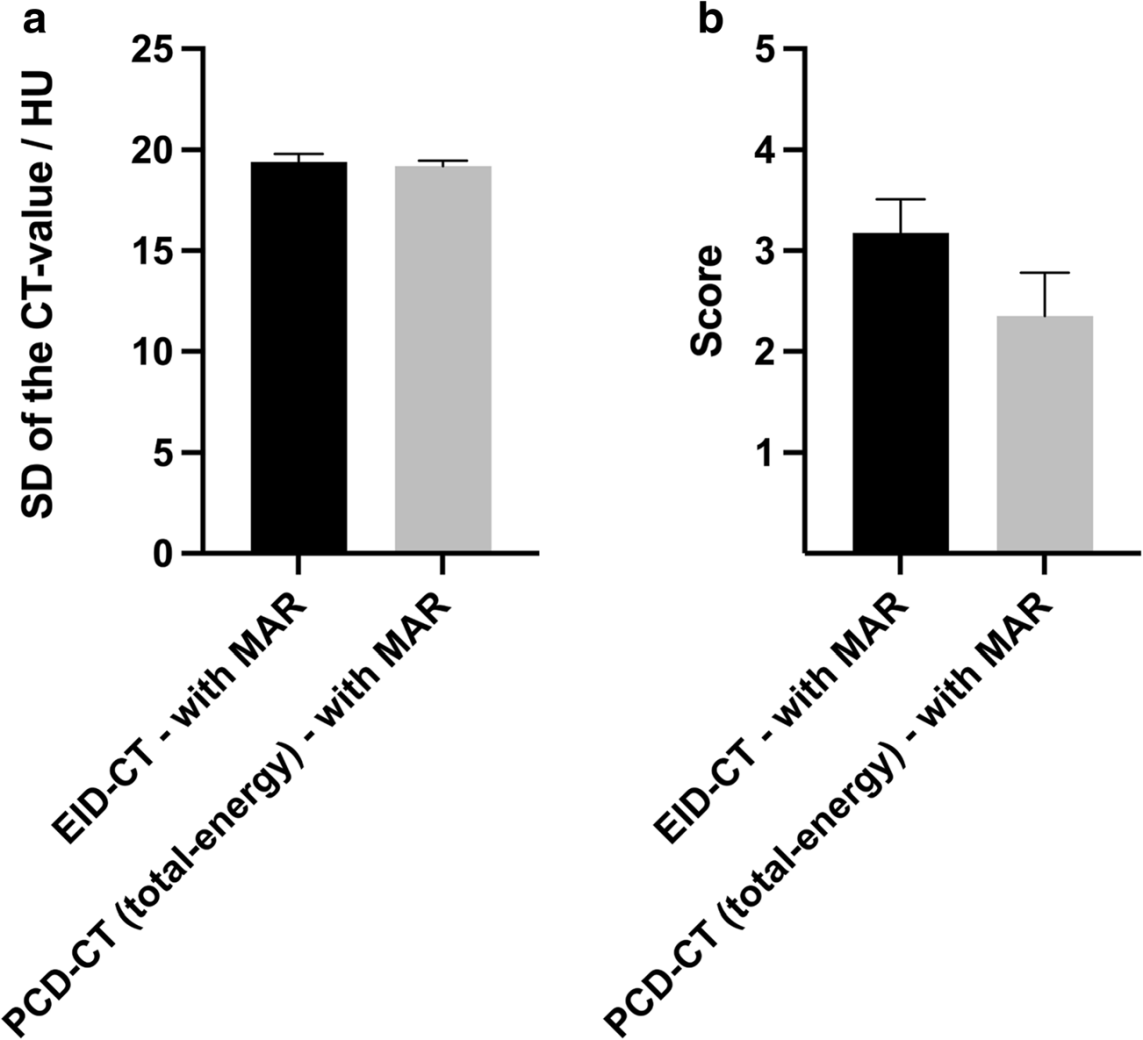
Illustration of the results of the quantitative (**a**) and qualitative (**b**) image analysis after metal artifact reduction (MAR). Quantitative analysis demonstrated a lower degree of coil-related artifacts for the photon-counting detector computed tomography (PCD-CT, total-energy images) compared to the energy-integrating computed tomography (EID-CT), achieving statistical difference in both analyses (*p* < 0.001, respectively). HU, Hounsfield units; SD, standard deviation. Bars: mean; whiskers: SD

A summary of the results of the quantitative image analysis is provided in Figs. 2 and 3 as well as Tables 2 and 4.

**Table 2:** Results of the quantitative and qualitative image analysis without metal artifact reduction

**Quantitative analysis**				
**PCD-CT** 25–120 keV			**EID-CT**	***p*** **value**
90.35 ± 6.28 HU			82.99 ± 7.89 HU	*p* < 0.001
**Qualitative analysis**				
**PCD-CT** 25–120 keV	**PCD-CT** 25–70 keV	**PCD-CT** 70–120 keV	**EID-CT**	***p*** **value**
4.65 ± 0.40	5.00 ± 0.00	4.18 ± 0.37	3.70 ± 0.64	*p* < 0.001

Summary of the results of the quantitative and qualitative image analysis of the PCD-CT and EID-CT scans without metal artifact reduction. For quantitative analysis, the Wilcoxon matched-pairs signed rank test and for qualitative analysis the Kruskal-Wallis test demonstrated a significant difference between the individual study groups, respectively. Quantitative results are provided as mean standard deviation (SD) ± SD of the Hounsfield units (HU) and qualitative results as mean score ± SD of the 5-point scale analysis. *p* values of the post hoc analysis of the qualitative results are demonstrated in Table 3

*PCD-CT*, photon-counting detector CT; *EID-CT*, energy-integrating detector CT

The Wilcoxon matched-pairs signed rank test showed a higher degree of coil-related artifacts for the total-energy PCD-CT images (25–120 keV; 90.35 ± 6.28 HU) compared to the EID-CT images (82.99 ± 7.89 HU) without MAR, using a standardized, donut-shaped ROI on the three central image slices (*p* < 0.001). After MAR, the artifacts were significantly lower in the total-energy PCD-CT images compared to the EID-CT images in quantitative analysis (*p* < 0.001).

The results of the qualitative image analysis are summarized in Figs. 2 and 3 as well as Tables 2, 3, and 4.

**Table 3:** Summary of the post hoc test for qualitative image analysis without metal artifact reduction

	PCD-CT 25–120 keV	PCD-CT 25–70 keV	PCD-CT 70–120 keV
PCD-CT 25–70 keV	*p* = 0.236	-	*p* < 0.001*
PCD-CT 70–120 keV	*p* = 0.048*	-	-
EID	*p* < 0.001*	*p* < 0.001*	*p* = 0.575

*p* values of the Dunn’s test for multiple comparisons using statistical hypothesis testing of the qualitative analysis

*PCD-CT*, photon-counting detector CT; *EID-CT*, energy-integrating detector CT

*indicates statistical significance

**Table 4:** Summary of the quantitative and qualitative image analysis after metal artifact reduction

**Quantitative analysis**		
**PCD-CT after MAR** 25–120 keV	**EID-CT after MAR**	***p*** **value**
19.20 ± 0.27 HU	19.41 ± 0.39 HU	*p* < 0.001
**Qualitative analysis**		
**PCD-CT after MAR** 25–120 keV	**EID-CT after MAR**	***p*** **value**
2.35 ± 0.43	3.18 ± 0.34	*p* < 0.001

Summary of the results of the quantitative and qualitative image analysis of the PCD-CT and EID-CT scans after metal artifact reduction. The Wilcoxon matched-pairs signed rank test demonstrated significantly lower artifacts for the PCD-CT images after metal artifact reduction compared to the EID-CT images

*HU*, Hounsfield units; *MAR*, metal artifact reduction; *PCD-CT*, photon-counting detector CT; *EID-CT*, energy-integrating detector CT

The Kruskal-Wallis test demonstrated a significant difference between the individual study groups of PCD-CT (total-energy PCD-CT, low-energy bin PCD-CT, and high-energy bin PCD-CT) and EID-CT without MAR (*p* < 0.001). Moreover, Dunn’s test for multiple comparisons revealed a higher degree of artifacts for the total-energy PCD-CT images (25–120 keV; 4.65 ± 0.40) compared to the high-energy bin PCD-CT images (70–120 keV; 4.18 ± 0.37; *p* = 0.048) and the EID-CT images (3.70 ± 0.64; *p* < 0.001) while there was no difference to the low-energy bin PCD-CT images (25–70 keV; 5.00 ± 0.00; *p* = 0.236), without MAR, respectively. Low-energy bin PCD-CT images (25–70 keV) presented a higher degree of artifacts compared to EID-CT (*p* < 0.001) while high-energy bin PCD-CT images (70–120 keV) presented a lower degree of artifacts compared to the low-energy bin PCD-CT images (25–70 keV, *p* < 0.001) and no difference compared to the EID-CT images, without MAR respectively. After MAR, the Wilcoxon matched-pairs signed rank test demonstrated a lower degree of artifacts in the total-energy PCD-CT images compared to the EID-CT images in qualitative analysis (2.35 ± 0.43 and 3.18 ± 0.34; *p* < 0.001).

Inter-reader and intra-reader reliability showed a strong agreement for the rating of the coil-related artifacts, respectively (inter-reader: *κ* = 0.816; range: 0.732–0.900; intra-reader: *κ* = 0.838; range: 0.759–0.918).

**Fig. 4:**
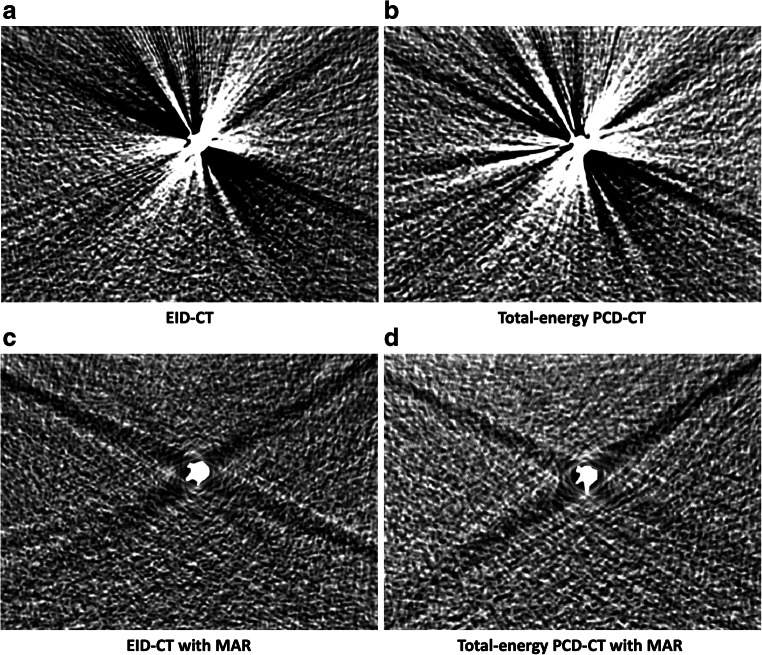
Representative images of the experimental in vitro model, resembling a coil-embolized aneurysm of the cerebral arteries, in energy-integrating computed tomography (EID-CT) and photon-counting detector computed tomography (PCD-CT, total-energy images) before (**a**: EID-CT; **b**: total-energy PCD-CT) and after metal artifact reduction (MAR; **c**: EID-CT; **d**: total-energy PCD-CT). Note the profound reduction of the coil-related artifacts. All images are provided in a standard CT brain imaging window (width: 40 Hounsfield units (HU) and level/center: 80 HU)

**Fig. 5:**
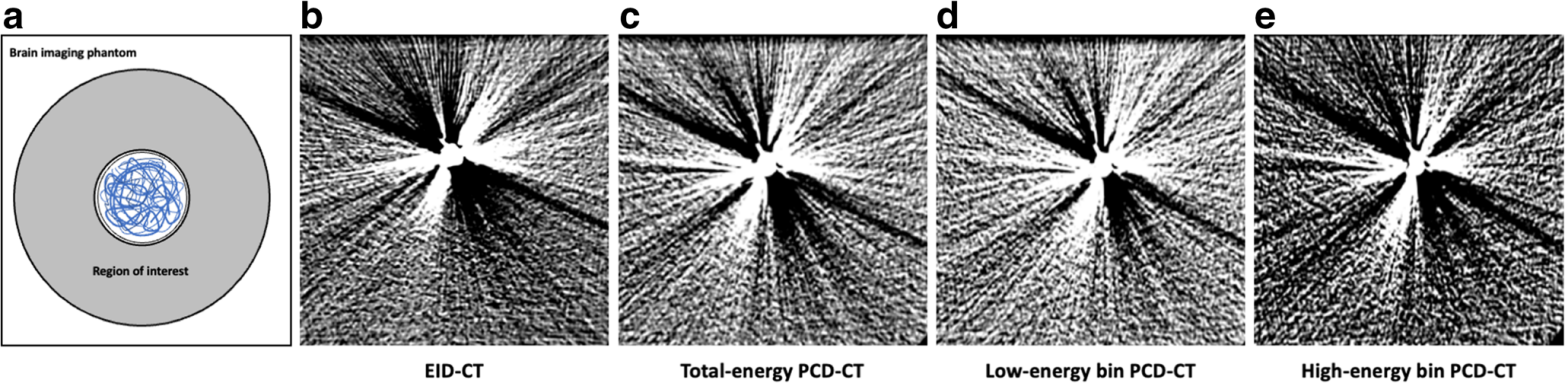
Schematic illustration of the quantitative analysis (**a**) and representative energy-integrating computed tomography (EID-CT) (**b**) and photon-counting detector computed tomography (PCD-CT) images (**c**–**e**) without metal artifact reduction in a standard CT brain imaging window (width: 40 Hounsfield units (HU) and level/center: 80 HU). For quantitative analysis, a donut-shaped region of interest was placed centrally to the coil-embolized in vitro model (**a**; coils: blue) on the three central image slices and the standard deviation of the HUs was calculated. Using a standard brain imaging protocol, EID-CT (**b**) and the high-energy bin PCD-CT images (**e**) demonstrated the lowest degree of artifacts while the low-energy bin PCD-CT images (**d**) showed the highest degree of artifacts with no difference compared to the total-energy PCD-CT images (**c**). Note the various image noise of the individual PCD-CT image reconstructions

Representative images of the PCD-CT and EID-CT scans before and after MAR are demonstrated in Figs. 4 and 5.

## Discussion

In this study, a systematic in vitro comparison of a standard brain imaging protocol in PCD-CT versus EID-CT with respect to metal artifacts induced by neurovascular platinum coils was conducted before and after MAR. Results of the quantitative and qualitative analysis demonstrated a higher degree of coil-related artifacts in the total-energy PCD-CT and low-energy bin PCD-CT images compared to the EID-CT images while there was no qualitative difference between the high-energy bin PCD-CT images and the EID-CT images without MAR, respectively. Further qualitative analysis of the PCD-CT scans without MAR revealed the lowest degree of coil-related artifacts for the high-energy bin PCD-CT images and the highest degree for the low-energy bin PCD-CT images while there was no difference between the total-energy PCD-CT and the low-energy bin PCD-CT images. After MAR, the artifacts were significantly lower in the total-energy PCD-CT images compared to the EID-CT images in quantitative and qualitative analysis. Thus, our in vitro findings suggest that the spectral information of the PCD-CT in combination with MAR has the potential to reduce coil-related metal artifacts more profoundly in cerebral imaging compared to EID-CT.

PCD-CT represents a promising new CT technique for clinical routine for various reasons, for example due to a reduction of radiation exposure [14]. To date there are only a limited number of reports available investigating human brain imaging and metal-related artifacts as well as options for MAR in PCD-CT [14–18, 28]. One recently published study by Pourmorteza et al performed an in vivo and in vitro comparison of PCD-CT and EID-CT and reported a greater gray-white matter contrast for the PCD-CT scans compared to conventional CT using a standard brain imaging protocol [28]. Moreover, they described that the image noise for the low-energy bin and the high-energy bin PCD-CT images was higher than that of the total-energy PCD-CT images because each bin contains only a subset of the detected photons. Comparing these image reconstructions, the low-energy bin PCD-CT images provided a good gray-white matter differentiation but were susceptible to beam-hardening while the high-energy bin PCD-CT images were less susceptible to beam-hardening but demonstrated a less adequate differentiation of gray and white matter. For this reason, we focused the quantitative analysis of our in vitro study on the total-energy PCD-CT images with the advantage to enable a more precise comparability between PCD-CT and EID-CT and at the same time having the best relationship between beam-hardening and noise. Furthermore, the degree of coil-related artifacts was assessed with a donut-shaped ROI placed on the three central image slices of the PCD-CT and EID-CT images. Compared to previous artifact-investigating PCD-CT studies which used circular ROIs or threshold-based segmentation for artifact assessment [15–18], the donut-shaped ROI allowed us to include all surrounding artifacts in a highly standardized procedure without taking the coils into account [23–25]. The calculation of the SD of the CT-values ensured that the coil-related artifacts, typically consisting of high- and low-attenuation areas [4, 5], might not be canceled out as it might be for the mean HUs.

Most studies investigating metal-related artifacts in PCD-CT focused on methods for MAR of orthopedic or dental implants [15–18]. For example, Zhou et al described a substantial decrease in artifacts of orthopedic implants using tin filtration in PCD-CT with a tube voltage of 140 kV [18] while Do et al demonstrated the lowest degree of artifacts of orthopedic implants for images acquired at 140 kV using high-energy bin image reconstruction [16]. Since a tube voltage of 140 kV and tin filtration would prevent a reliable differentiation of the gray and white matter on non-contrast-enhanced cerebral CT images, image acquisition of the present study focused on a tube voltage of 120 kV without tin filtration. Comparing the PCD-CT and EID-CT images, quantitative analysis demonstrated a significantly lower degree of coil-related artifacts for EID-CT without MAR. Similar findings were demonstrated for qualitative analysis with a higher degree of artifacts for the low-energy bin PCD-CT and total-energy PCD-CT images compared to EID-CT, while there was no statistical difference between the EID-CT images and the high-energy bin PCD-CT images without MAR, respectively. These results are in accordance with the findings of Pourmorteza et al [28] and Do et al [16] that the high-energy bin images are less susceptible to metal-related artifacts. A possible reason for this could be that the effective energy of the high-energy bins is greater and thus the attenuation coefficient is significantly smaller in PCD-CT compared to EID-CT. However, the low-energy x-ray photons are primary responsible for the soft tissue contrast. To combine the advantages of the high-energy bin images and the low-energy bin images, further MAR was performed for the total-energy PCD-CT images and compared to the EID-CT images. So far, there is no commercial software for MAR in PCD-CT images available. In order to provide a reliable comparability between the PCD-CT and EID-CT images and due to the limited complexity of the used in vitro model, linear interpolation MAR was applied to both CT image datasets [21]. Further MAR procedures such as the photon-counting normalized metal artifact reduction (PCNMAR) by Byl et al may provide a more reliable MAR for clinical PCD-CT images of higher complexity [15]. However, the PCNMAR was specifically designed for PCD-CT imaging data and therefore not suitable for the present study. The present in vitro results demonstrated that metal artifacts can be reduced more profoundly in the PCD-CT images compared to the EID-CT images in quantitative and qualitative analysis. Thus, the spectral information of the PCD-CT has the potential to provide an improved option for MAR in cerebral CT imaging of patients after endovascular platinum coil-embolization. However, despite reaching statistical significance, the subjective differences between the PCD-CT and EID-CT images after MAR are small and the clinical impact on the advantage of the present findings is unclear. Certainly, there will be no major impact on the detection of large cerebral pathologies, but there may be a minor advantage for the detection of small pathologic lesions, which need to be further investigated in clinical studies.

We acknowledge that this study has several limitations. Since the present study used a brain imaging phantom, the transferability of an in vitro model to the human brain is limited and future in vivo studies are necessary. Moreover, the mixture of contrast medium and saline within the brain imaging phantom may result in a different attenuation depending on the specific energy of the photons used for imaging, which might lead to certain bias in quantitative and qualitative analysis. Due to cost-factors, our model contained only a defined number of neurovascular coils while a variable number of coils might have further influence on the degree of artifacts. Only the CT brain imaging settings of our institution have been applied while other institutions might use different settings. Moreover, only the Macro imaging mode was used for PCD-CT imaging; thus, other available PCD-CT imaging modes might lead to different results. Also the intrinsically available spectral information of PCD-CT has not been exploited in this study.

## Conclusion

PCD-CT constitutes a promising new technique for future CT imaging in clinical routine. The results of the present study demonstrated no difference in the degree of platinum coil–related artifacts between the high-energy bin PCD-CT images and the conventional EID-CT images while low-energy bin PCD-CT and total-energy PCD-CT images both showed a higher degree of artifacts, without MAR, respectively. However, linear interpolation MAR was able to reduce the coil-related artifacts in the total-energy PCD-CT images with differences compared to the EID-CT images in both analyses. Thus, PCD-CT in combination with MAR has the potential to provide an improved option for reduction of coil-related artifacts in cerebral imaging in this in vitro study.

## Abbreviations

CT: Computed tomography
DKFZ: German Cancer Research Center
EID-CT: Energy-integrating detector CT
HU: Hounsfield units
L: Window level/center
MAR: Metal artifact reduction
MITK: Medical Imaging Interaction Toolkit
PCD-CT: Photon-counting detector CT
PCNMAR: Photon-counting normalized metal artifact reduction
ROI: Region of interest
SD: Standard deviation
W: Window width

## Declarations

### Guarantor

The scientific guarantor of this publication is Dominik Vollherbst.

### Statistics and biometry

No complex statistical methods were necessary for this paper.

### Methodology

• Prospective

• Experimental

• Multicenter study
